# Support vector regression-based QSAR models for prediction of antioxidant activity of phenolic compounds

**DOI:** 10.1038/s41598-021-88341-1

**Published:** 2021-04-22

**Authors:** Ying Shi

**Affiliations:** Department of Chemistry, Baotou Teachers’ College, Baotou, 014030 China

**Keywords:** Biochemistry, Chemical biology, Molecular biology, Molecular medicine, Chemistry

## Abstract

The Support vector regression (SVR) was used to investigate quantitative structure–activity relationships (QSAR) of 75 phenolic compounds with Trolox-equivalent antioxidant capacity (TEAC). Geometric structures were optimized at the EF level of the MOPAC software program. Using Pearson correlation coefficient analysis, four molecular descriptors [n(OH), Cosmo Area (CA), Core-Core Repulsion (CCR) and Final Heat of Formation (FHF)] were selected as independent variables. The QSAR model was developed from the training set consisting of 57 compounds and then used the leave-one-out cross-validation (LOOCV) correlation coefficient to evaluate the prediction ability of the QSAR model. Used Artificial neural network (ANN) and multiple linear regression (MLR) for comparing. The RMSE (root mean square error) values of LOOCV in SVR, ANN and MLR models were 0.44, 0.46 and 0.54. The RMSE values of prediction of external 18 compounds were 0.41, 0.39 and 0.54 for SVR, ANN and MLR models, respectively. The obtained result indicated that the SVR models exhibited excellent predicting performance and competent for predicting the TEAC of phenolic compounds.

## Introduction

Phenolic compounds are natural products and can be extracted easily from many plants^1^. They show extensive biological activities such as anti-hepatotoxic^2^, antitumor^3^, anti-inflammatory^4,5^ and antioxidant activity^6–8^. Among them, antioxidant activity depends mainly on the structure^9–11^, so numerous researcher establish many quantitative structure–activity relationships (QSAR) models to investigated the antioxidant activity of flavonoids and interpret the relationship between phenolic compounds structure and their antioxidant activity^12–16^, the optimized QSAR model is helpful for researchers to design and synthesize antioxidants. Because of the complex relationship between phenolic compounds structure and antioxidant activity, simple linear models are insufficient to explain the effect of structural parameters on antioxidant activity^17,18^. Therefore, it is essential to use machine learning algorithms such as multiple linear regression (MLR), artificial neural networks (ANNs) to improve the predictability of QSAR^19,20^. Djeradi et al. have used Fukui indices and MLR method for prediction antioxidant activity of DPPH test of 24 flavonoids, the square of correlation coefficient (R^2^) of their model is 0.816^21^. Cerit et al. have used a multilayer perceptron (MLP) ANN to predict the effect of ferric ion on the antioxidant capacity of phenolic, the average errors of prediction of the training set and validation sets are 8.5 and 10.1%^22^. Li et al. have used MLP-ANN model to predict the antioxidant activity of polysaccharides in DPPH test and used sensitivity analysis to interpret the effect of the input variables on the target values^23^. Petar et al. and Fatemi et al. have used ANN and MLP-ANN QSAR models to evaluate the contribution of the quantum mechanical molecular descriptors to the Trolox-equivalent antioxidant capacity (TEAC) in an optimized ANN model^19,24^. Although the prediction accuracy of ANN is higher than MLR, most of the current ANN methods used to predict antioxidant activity are more like a black box that has overfitting risk and lead to unreliable predictions. Besides, it comprises a single hidden layer with an arbitrary activation function that must be bonded.

In addition to the above algorithm, support vector regression (SVR) is a useful machine learning algorithms that can be used to solve linear and nonlinear problems^25^, especially for small sample sizes. It has been proved to be suitable for the QSAR analyses of flavonoids^26^, drug activity prediction and design^27^. For instance, Minaoui et al. have used support vector regression to investigate the relationship between structure and activity of 38 cyclicurea derivatives, inhibiting HIV protease. In their work, each molecule is described by four descriptors, and the parameters of the SVR model are optimized by grid optimization. Then they compared the R^2^ and RMSE values of the prediction results of MLR, ANN, and SVR methods. The obtained results show that the SVR model has better qualities and better generalization capabilities than other methods. By evaluating the contribution of the molecular descriptors to the model established by the SVR, they also found that the molar volume and dipole moment parameters of the compounds take the most relevant part in the molecular description and controlling the biological activity of cyclic-urea derivatives^28^.

In addition to modelling methods, a reliable QSAR models also need to select appropriate variables, the QSAR models usually using topological and quantum mechanical parameters. Density functional theory (DFT) is an accurate but time consuming method for calculating electronic structure parameters^29^. While the Semi-empirical Hamiltonians method can obtain reliable molecular parameters for building QSAR models in a more time-efficient way^30^, especially when there is a lack of experience in selecting descriptors.

This study use Semi-empirical Hamiltonians (PM7, MOPAC 2016) to obtain molecular descriptors, then use Pearson correlation coefficient analysis for selecting molecular descriptors, then use the SVR method to develop a QSAR model to predict the antioxidant activity of 75 phenolic compounds. For comparing the prediction ability, ANN and MLR methods are used to build the QSAR models, too.

## Materials and methods

### Methods

#### Support vector regression (SVR)

As a statistical learning method, SVR uses a kernel function (including the linear kernel function (LKF), the polynomial kernel function (PKF), and the radial basic function (RBF) kernel function) to map the vectors into a higher dimensional feature space. By introducing an alternative loss function and kernel function, SVR can be applied to linear regression of the target variable in this space. For detailed information on the optimal regression function and related Lagrangian expressions, see Refs.^20,31^.

#### Leave one out cross-validation (LOOCV)

LOOCV process: first, each sample in the training dataset will be removed, and then use the remaining samples to build a model and predict the target value of the removed sample. In this work, the reliability was evaluated by LOOCV, and used tenfold-cross-validation (tenfold-CV) to search for the optimal kernel function type and corresponding parameters^32,33^.

#### Sensitivity analysis (SA)

Sensitivity analysis is often used to obtain the influence degree of variables on the target variable. SA can provide an effective method to characterize the uncertainties between characteristic parameters and models^34,35^. Based on the straightforward characteristics of SA, it was used in this work to explain the influence of parameters on TEAC.

#### Model accuracy

To obtain appropriate kernel function and capacity parameter C, insensitive loss function ε and the corresponding parameters gamma g of the kernel function in this computation, the least root mean square error (RMSE) and correlation coefficient R were used as the evaluation criterion^20^. RMSE is defined as follows:

1 $${\mathrm{RMSE}}=\sqrt{\frac{\sum_{i=1}^{n}{({p}_{i}-{e}_{i})}^{2}}{n}}$$

where *n* is the number of total samples, *e*_i_ and *p*_i_ are the experimental value and the predicted value of sample i, respectively. Generally, the smaller RMSE means the better expected predictive ability.

The prediction power of the training set and test set also validated by statistical parameters of correlation coefficient (Q^2^)^36,37^, Q^2^ is defined as

2 $${Q}^{2}=1-\frac{\sum {({e}_{i}-{p}_{i})}^{2}}{\sum ({{e}_{i}-{p}_{mean})}^{2}}$$

All the methods calculated on the ExpMiner Software (version 2.1.1.0, Laboratory of Materials Data Mining, Department of Chemistry, College of Sciences, Shanghai University, China).

### Data sets

#### 75 phenolic compounds and TEAC values

The antioxidant activity (TEAC values, ABTS^·+^ assay) of 75 phenolic compounds were obtained from a study by Cai et al.^38^, The data set was randomly divided into the training set (57 phenolic compounds, ~ 75%) and the testing set (18 phenolic compounds, ~ 25%).

#### Molecular descriptors

The molecular descriptors of each phenolic compound were calculated by MOPAC software with EF geometry optimization and PM7 Semi-empirical Hamiltonians (MOPAC2016, J.J.P. Stewart, Stewart Computational Chemistry, Colorado Springs, CO, USA).

The name and molecular descriptors of phenolic compounds were given in Table S1.

## Results

### Descriptor selection and data set

Due to the existence of irrelevant or redundant features redundancy of the parameters, it is necessary to select the parameter most relevant to the target variable, especially when the sample set is small. The purpose of feature selection is to select a variables subset of *n* features from the set of *m* obtained variables (*n* < *m*) without significantly reducing the predictive ability of the model^27^. In this work, the total number of calculated molecular descriptors was eight. Used Pearson correlation selection modules to select descriptors (ExpMiner software), then the most significant three descriptors were selected. Since n(OH) is a critical variable and easy to get, added it to the variables. Finally, a total of four descriptors were chosen to construct the QSAR models, the descriptions of descriptors are shown in Table 1.

**Table 1:** Molecular descriptors involved in the QSAR models.

Molecular descriptor	Description
n(OH)	Number of OH groups
CA	Cosmo area
CCR	Core–core repulsion
FHF	Final heat of formation

### Grid-search for parameter optimization

In the modeling process, the parameters of the model were selected by grid search method, and the parameters of the lowest RMSE were found with three different kernel functions (RBF, PKF and LKF kernel function), that is, the optimal parameters.

**Figure 1:**
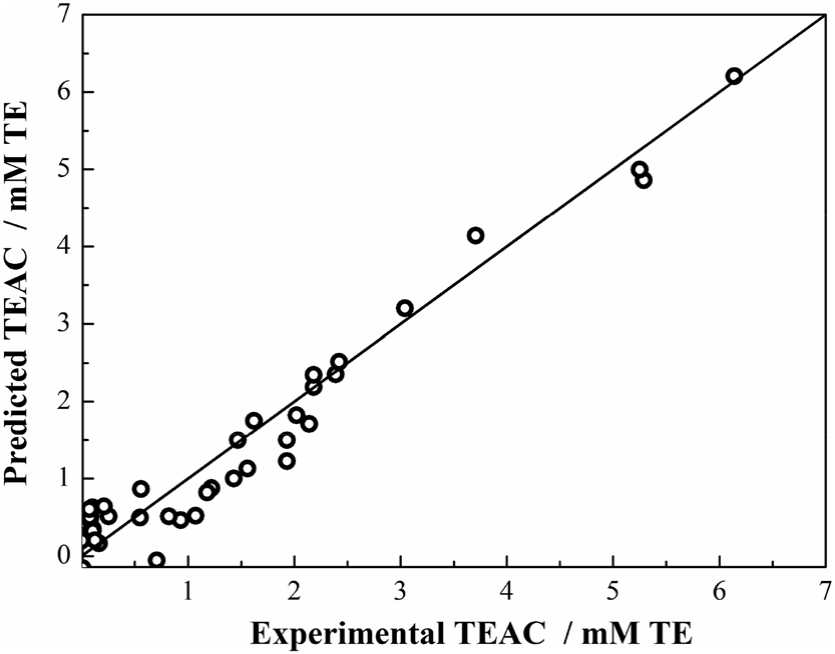
Correlation of experimental and predicted activity of agonists using Eq. (3).

By tenfold cross-validation in the grid-search process, RMSE values were calculated with capacity parameter (*C, C* = 1–500, step = 10) and e-insensitive loss function parameter (*ε*, *ε* = 0.01–0.1, step = 0.01)) with LKF and PKF, *C* (*C* = 1–500, step = 10), *ε* (*ε* = 0.01–0.1, step = 0.02) and Gamma (*g*, *g* = 0.5–1.5, step = 0.1) with RBF kernel function. The minimum RMSE values of RBF, PKF and LKF kernel function were 0.41, 0.45 and 0.50, respectively (see Supporting Information Fig. S1). Hence, the optimal SVR model is SVR-RBF kernel function with *C* = 121, *ε* = 0.07, *g* = 0.6 and the corresponding equation is:

3 $${\text{TEAC}} = \sum ({{\alpha}}_{{\text{i}}} - {{{\alpha}}_{{\text{i}}}}^{*} ) \times {\text{exp}}[ - 0.{6} \times (|{\text{X}} - {\text{X}}_{{\text{i}}} *|)^{{2}} ] + \, 0.{758}$$

where α_i_ − α_i_* is the Lagrange coefficient corresponding to the 24 support vectors, the correlation coefficient between the predicted value and the experimental value is 0.967, as shown in Fig. 1.

### LOOCV result of SVR-QSAR model

**Figure 2:**
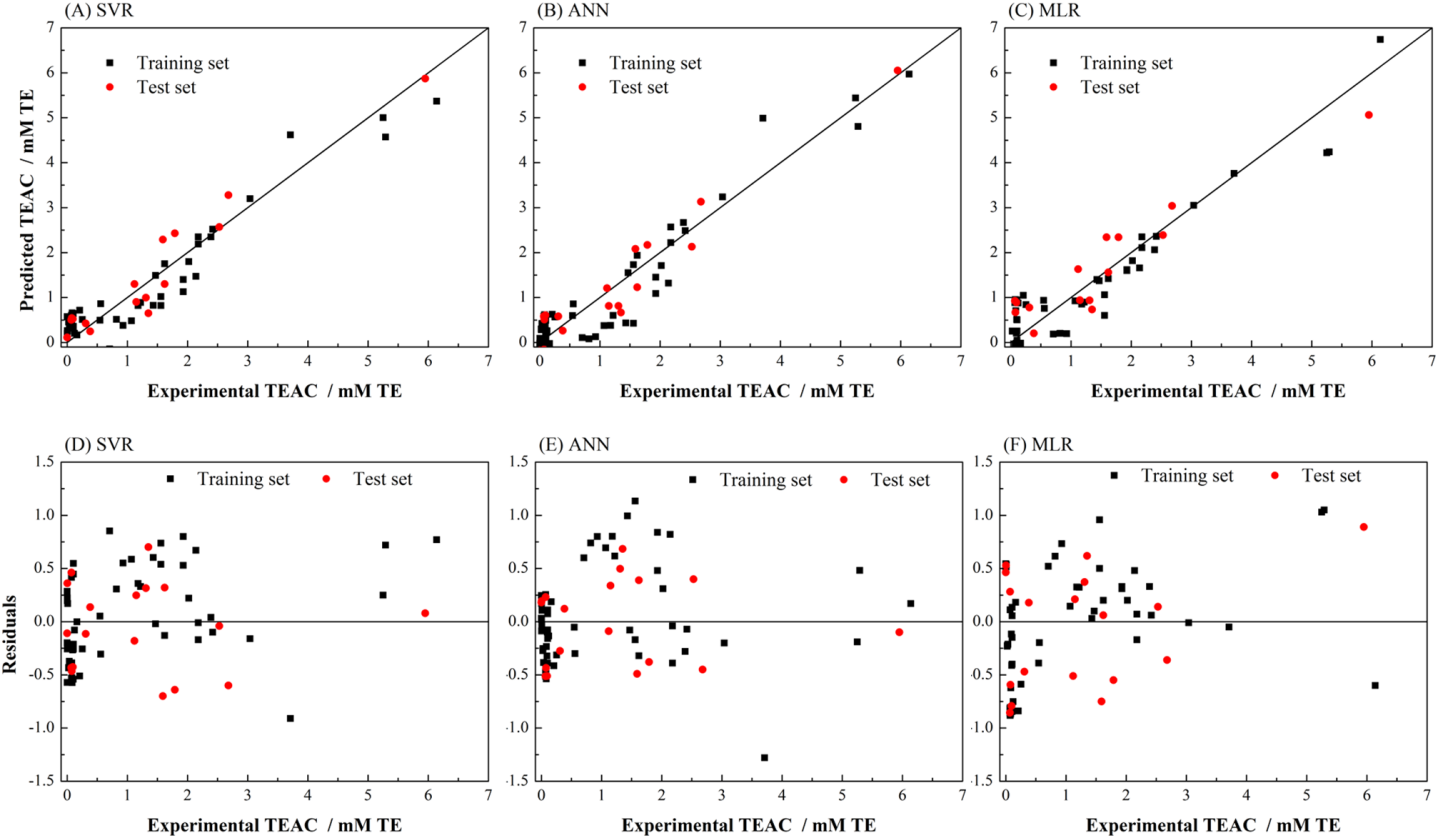
Experimental vs predicted activities of TEAC in LOOCV test and external test set of (**A**) SVR (using RBF kernel), (**B**) ANN, and (**C**) MLR. The plot of predicted residuals vs experimental values of TEAC of (**D**) SVR, (**E**) ANN and (**F**) MLR.

LOOCV was used to verify the reliability of the predictive ability of the QSAR Model. The same parameters were used to model with SVR, ANN and MLR to predict the TEAC values of 57 phenolic compounds (training set), then used the LOOCV method to examine their respective generalization capabilities (Fig. 2). The experimental values, predicted values of the training set and the test set are given in Table 2. The correlation coefficient (R^2^) between the predicted TEAC values and the experimental TEAC values of LOOCV are 0.904, 0.897 and 0.856 in SVR, ANN and MLR models. The results of Q^2^ obtained by the three modelling methods are similar to those of R^2^ (Table 3). The RMSE value of prediction of the test set in SVR is slightly higher than that of ANN, but the SVR model has the lowest predict RMSE of LOOCV, it is suggested that the generalization ability of SVR was superior to ANN and MLR in this work. From the results of residual, SVR is relatively stable in the whole data range, but the residuals of ANN and MLR are larger when the TEAC values are near 1.5 and 0.

**Table 2:** Predicted TEAC with different methods using LOOCV (No. 1–57) and test set (No. 58–75).

No.	Experimental TEAC/mM TE	Predicted TEAC/mM TE		
		SVR	ANN	MLR
1	1.56	0.821	0.425	0.603
2	0.93	0.379	0.129	0.197
3	0.82	0.513	0.08	0.204
4	0.007	0.259	0.04	− 0.508
5	1.22	0.89	0.603	0.897
6	0.037	0.41	0.422	0.25
7	0.025	0.456	0.298	0.256
8	0.028	0.462	0.281	0.251
9	0.092	0.359	0.414	0.209
10	0.005	0.261	− 0.165	− 0.518
11	5.29	4.57	4.808	4.24
12	3.71	4.62	4.99	3.76
13	3.04	3.20	3.24	3.05
14	2.39	2.35	2.67	2.06
15	2.02	1.80	1.71	1.82
16	2.18	2.19	2.22	2.11
17	1.56	1.02	1.73	1.06
18	0.707	− 0.145	0.107	0.186
19	2.42	2.52	2.49	2.36
20	1.93	1.40	1.45	1.60
21	1.43	0.826	0.435	1.40
22	2.18	2.35	2.57	2.35
23	0.081	0.608	0.619	0.948
24	1.47	1.49	1.55	1.37
25	0.083	0.656	0.318	0.705
26	0.003	− 0.195	0.09	− 0.536
27	0.098	0.358	0.195	0.509
28	0.104	0.358	− 0.006	0.503
29	0.000	− 0.284	− 0.246	− 0.544
30	0.101	0.642	0.491	0.947
31	0.072	− 0.344	− 0.182	− 0.04
32	0.005	− 0.217	− 0.026	− 0.537
33	5.25	5.00	5.44	4.22
34	6.14	5.37	5.97	6.74
35	2.14	1.47	1.32	1.66
36	1.62	1.75	1.94	1.42
37	0.558	0.863	0.858	0.756
38	0.002	0.574	− 0.03	− 0.503
39	1.18	0.821	0.378	0.854
40	0.164	0.166	− 0.023	− 0.018
41	0.001	0.2	− 0.021	− 0.506
42	0.253	0.509	0.566	0.841
43	0.104	− 0.443	0.182	0.048
44	0.209	0.719	0.624	1.048
45	1.93	1.13	1.09	1.62
46	1.07	0.483	0.376	0.925
47	0.548	0.496	0.6	0.937
48	0.076	0.486	0.587	0.884
49	0.069	0.501	0.582	0.928
50	0.068	0.511	0.528	0.886
51	0.077	0.465	0.537	0.905
52	0.076	0.507	0.517	0.955
53	0.072	0.606	0.512	0.953
54	0.105	− 0.341	0.031	− 0.029
55	0.009	− 0.162	− 0.098	− 0.525
56	0.105	0.318	0.263	0.254
57	0.124	0.204	0.259	0.876
58	1.31	0.995	0.812	0.938
59	1.15	0.903	0.812	0.94
60	5.95	5.87	6.05	5.06
61	2.68	3.28	3.13	3.04
62	1.59	2.29	2.08	2.34
63	1.12	1.30	1.21	1.63
64	1.79	2.43	2.17	2.34
65	0.001	0.110	− 0.179	− 0.463
66	0.097	0.524	0.608	0.889
67	0.077	0.543	0.511	0.671
68	2.53	2.57	2.13	2.39
69	1.35	0.649	0.666	0.732
70	0.383	0.246	0.261	0.204
71	0.003	− 0.357	− 0.191	− 0.527
72	0.308	0.423	0.582	0.778
73	1.62	1.30	1.23	1.56
74	0.068	0.501	0.581	0.924
75	0.073	− 0.388	− 0.158	− 0.208

**Table 3:** RMSE and the squared correlation coefficient (R^2^ and Q^2^) of antioxidant activity prediction in LOOCV and test set of three models (SVR, ANN and MLR).

	SVR	ANN	MLR
**LOOCV**			
RMSE	0.440	0.464	0.539
R^2^	0.904	0.897	0.856
Q^2^	0.903	0.892	0.855
**Test set**			
RMSE	0.410	0.386	0.536
R^2^	0.925	0.931	0.861
Q^2^	0.917	0.927	0.859

### Sensitivity analysis (SA) of SVR-QSAR model

**Figure 3:**
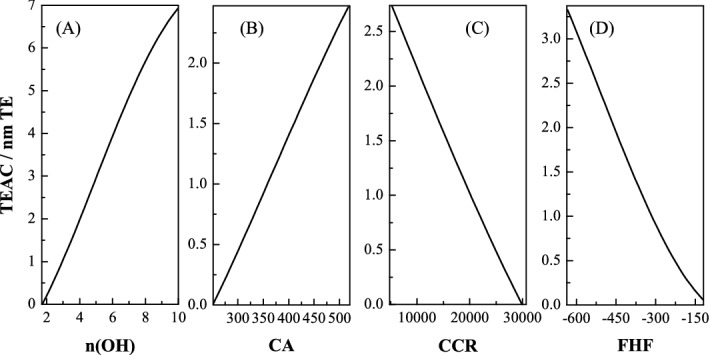
TEAC vs n(OH), CA, FHF and CCR by SA.

Sensitivity analysis was used for analysis the correlation of molecular descriptors with TEAC, From Fig. 3, it can be suggested that the value of TEAC increased with the increase of n(OH) and CA, decreased with the increase of CCA and FHF. Further analysis showed that the order of the descriptors' influence on TEAC in descending is n(OH) > CA > FHF > CCR.

## Discussion

### The QSAR model based on SVR

In LOOCV test, SVR is superior to ANN and MLR. In the test set, the prediction ability of SVR is better than that of MLR, and is basically equal to that of ANN. From the result of residual error, SVR also shows good stability of prediction ability. However, the selection of kernel function and the optimization of parameters in SVR modelling were more time-consuming than ANN and MLR. There may be other more suitable parameters outside the scope of the gridding parameter selection. However, SVR is still a kind of regression method with higher accuracy, and it can be used for the establishment and analysis of QSAR models. In the future, further algorithm optimization can be carried out to shorten the kernel function selection and grid parameter selection process.

### The relationship between TEAC and molecular descriptors

Sensitivity analysis in the SVR-QSAR model had shown that four characteristic parameters significantly affect the TEAC of phenolic compounds (Fig. 3).

Based on the hydrogen transfer mechanisms in the antioxidant process, an increase in hydroxyl groups means more hydrogen atoms that can be transferred, thereby increasing the TEAC value^39^. Core-Core Repulsion is relevant to molecular size, the shorter bond length means the larger CCR value. Some studies have shown that changes in CCR value affect the rate of intermolecular reactions^40,41^. In this work, the lower CCR value was beneficial to increased antioxidant activity of phenolic compounds. As for the Final heat of formation value, which reflects the stability of the molecule, a more stable molecule lead to lower antioxidant activity. The effect of Cosmo Area on TEAC is opposite to that of Core-Core Repulsion, large Cosmo Area lead to better antioxidant activity.

Compare the previous similar research based on the DFT parameters (minimum bond dissociation enthalpy (BDE(min)), HOMO and LUMO energies of the neutral species, ionization potential (IP), and dipole moment of the neutral species)^42,43^. This work reveals the potential modelling and prediction capabilities of the model use parameters obtained by Semi-empirical Hamiltonians, which is more time-efficient.

### Applicability domain analysis

If a QSAR model is to be used for screening new compounds, the domain of application of this QSAR model must be defined^28^. The leverage *h*_*i*_ of a compound can be used for judging the compound is in the domain or not, which is defined as follows:

4 $${h}_{i}={x}_{i}^{T}{({x}^{T}x)}^{-1}{x}_{i} (i=1\ldots n)$$

where *x*_*i*_ is the descriptor vector of the considered compound and *x* is the descriptor matrix derived from the training set. The superscript *T* refers to the transpose of the matrix/vector. The warning leverage h* is fixed at 3(*p* + 1)/*n*, where *n* is the number of training compounds and *p* is the number of model parameters. In this model, the value of h* is 0.263. A leverage greater than the warning leverage h* means that the predicted response may not be reliable.

**Figure 4:**
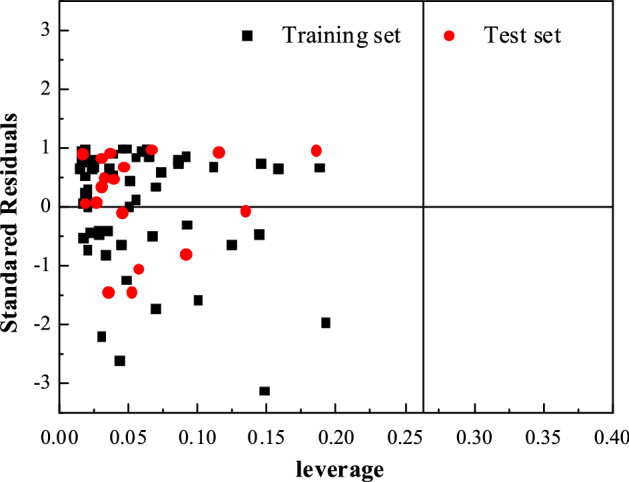
The plot of standardized residuals vs leverage, with a warning leverage of h* = 0.263.

The plot of leverage and standard residuals for the SVR-QSAR model is shown in Fig. 4. As shown in the Williams plot (Fig. 4), *h*_*i*_ values of all the compounds in the training and test sets are lower than the warning value (h* = 0.263). The training set has great representativeness, and none of the compounds is particularly influential in the model space. The standardized residual of compound number 12 was slightly larger than three standard deviation units (3* s*), which may be due to its different antioxidant activity mechanism.

## Conclusions

In this study, an SVR-QSAR model of 75 phenolic compounds TEAC values was developed. The Pearson correlation coefficient method was employed in the parameter selection process in QSAR model development. Satisfactory prediction results were obtained using four parameters calculated by Semi-empirical Hamiltonians PM7. Although the SVR-QSAR model shows good stability of prediction ability, the SVR still has some shortcomings, such as selecting kernel function and the optimization of modeling parameters were more time-consuming than ANN and MLR. There may be other more suitable parameters outside the scope of the gridding parameter selection. Continuous optimization algorithms can be used in the future to reduce the time-consuming of the SVR-modelling process. Gupta et al. have done a series of work in this field^44–47^, they proposed a new unconstrained convex minimization problem formulation equivalent to the Lagrangian dual of the 2-norm twin support vector regression (TSVR), using the proposed formulation on synthetic and real-world datasets demonstrates a significant increase in learning speed with better accuracy in performance in accordance with the classical support vector regression and twin support vector regression^47^. Therefore, in order to obtain a better and faster SVR model in the subsequent work, it is necessary to continuously optimize the algorithm.

## Supplementary Information

Supplementary Information.
