# Supplementary material for: Support vector regression-based QSAR models for prediction of antioxidant activity of phenolic compounds
Source: Sci Rep. 2021 Apr 22;11:8806. doi: 10.1038/s41598-021-88341-1 (PMC8062522; doi:10.1038/s41598-021-88341-1)
Supplement: Supplementary file 1 — Supplementary Information. [file 41598_2021_88341_MOESM1_ESM.docx]

*Supporting Information*

**Support Vector Regression-Based QSAR Models for Prediction of Antioxidant Activity of Phenolic Compounds**

Ying Shi *

Department of Chemistry, Baotou Teachers’ College

shiying@bttc.edu.cn

*To whom correspondence should be addressed.

E-mail: shiying@bttc.edu.cn

**Table** S1. The name and molecular descriptors of 75 phenolic compounds (Training set: No.1-57 and test set: No.58-75).

| No | Name of phenolic compounds | n(OH)^a^ | CA^b^ | CCR^c^ | FHF^d^ |
| --- | --- | --- | --- | --- | --- |
| 1 | Chlorogenic acid | 2 | 338.43 | 29898.76 | -361.558 |
| 2 | *o*-Coumaric acid | 1 | 192.72 | 8046.221 | -92.1423 |
| 3 | *m*-Coumaric acid | 1 | 195.86 | 7841.646 | -91.0926 |
| 4 | *trans*-Cinnamic acid | 0 | 185.68 | 6618.601 | -52.7815 |
| 5 | 2,4-Hydroxybenzoic acid | 2 | 167.67 | 7513.394 | -162.505 |
| 6 | *o*-Hydroxybenzoic acid | 1 | 157.61 | 6301.139 | -114.894 |
| 7 | *m*-Hydroxybenzoic acid | 1 | 160.34 | 6144.857 | -102.596 |
| 8 | *p*-Hydroxybenzoic acid | 1 | 161.1 | 6110.296 | -112.43 |
| 9 | Vanillic acid | 1 | 186.94 | 8753.783 | -142.642 |
| 10 | Benzoic acid (ck) | 0 | 149.86 | 5005.668 | -58.4815 |
| 11 | (−)-Epicatechin gallate (ECG) | 7 | 386.07 | 42966.02 | -258.041 |
| 12 | (−)-Epigallocatechin (EGC) | 6 | 285.69 | 24240.86 | -265.823 |
| 13 | (+)-Catechin | 5 | 281.11 | 22051.45 | -222.699 |
| 14 | Quercetin-3-glucoside | 4 | 396.89 | 48245.19 | -448.23 |
| 15 | Quercetin-3-rutinoside | 4 | 473.54 | 80545.21 | -633.409 |
| 16 | Quercetin-3-rhamnoside | 4 | 380.99 | 46321.67 | -409.975 |
| 17 | Quercetin-3-glucoside-7-rhamnoside | 3 | 516.99 | 75198.75 | -627.98 |
| 18 | Flavonol | 1 | 254.31 | 15737.3 | -63.09 |
| 19 | Butein | 4 | 287.82 | 17726.88 | -150.248 |
| 20 | Sappanchalcone | 3 | 301.9 | 19933.19 | -147.079 |
| 21 | Carthamin | 3 | 370.34 | 45278.8 | -368.698 |
| 22 | Luteolin | 4 | 281.43 | 19809.13 | -185.876 |
| 23 | Chrysin | 2 | 262.76 | 16438.28 | -98.445 |
| 24 | Luteolin-7-glucoside | 3 | 404.38 | 44025.82 | -399.513 |
| 25 | Apigenin-7-glucoside | 2 | 390.25 | 41632.25 | -367.463 |
| 26 | Flavone (ck) | 0 | 244.45 | 13102.74 | -8.62175 |
| 27 | Naringin | 2 | 482.54 | 71970.84 | -586.839 |
| 28 | Hesperidin | 2 | 477.08 | 76753.95 | -614.575 |
| 29 | Flavanone (ck) | 0 | 248.69 | 13809.6 | -29.1589 |
| 30 | Daidzein | 2 | 262.54 | 16172.21 | -99.5425 |
| 31 | Daidzein-7-glucoside | 1 | 379.88 | 38498.26 | -325.578 |
| 32 | Isoflavone (ck) | 0 | 242.27 | 13173.84 | -7.72333 |
| 33 | Catechin-3-*O*-gallate | 7 | 383.51 | 43937.33 | -354.517 |
| 34 | Procyanidin B-1 (dimer) | 10 | 430 | 73942.91 | -451.127 |
| 35 | Resveratrol | 3 | 261.74 | 13144.51 | -76.6924 |
| 36 | Piceatannol-3'-glucoside | 3 | 403.21 | 36479.04 | -335.127 |
| 37 | Resveratrol-4'-glucoside | 2 | 396.22 | 33148.3 | -293.492 |
| 38 | *trans*-Stilbene | 0 | 233.35 | 9027.798 | 58.81523 |
| 39 | Bisdemethoxycurcumin | 2 | 347.18 | 20659.71 | -90.2252 |
| 40 | Esculetin-6-glucoside | 1 | 315.23 | 29127.61 | -341.699 |
| 41 | Coumarin (ck) | 0 | 168.72 | 6726.772 | -37.0672 |
| 42 | Matairesinol | 2 | 367.5 | 29539.04 | -209.169 |
| 43 | Arctigenin | 1 | 388.23 | 31621.13 | -203.206 |
| 44 | Magnolol (neolignans) | 2 | 310.97 | 18465.29 | -28.6067 |
| 45 | Purpurin | 3 | 240.94 | 17135.32 | -156.951 |
| 46 | Alizarin | 2 | 234.81 | 15219.34 | -106.555 |
| 47 | Quinizarin | 2 | 232.8 | 15377.14 | -112.675 |
| 48 | Rhein | 2 | 265.36 | 19787.42 | -184.368 |
| 49 | Chrysophanol | 2 | 252.12 | 16987.43 | -126.206 |
| 50 | Physcion | 2 | 280.68 | 20392.28 | -168.581 |
| 51 | Aloe-emodin | 2 | 262.33 | 18647.47 | -150.496 |
| 52 | 1,5-Dihydroxyanthraquinone | 2 | 234.78 | 15333.88 | -104.837 |
| 53 | 2,6-Dihydroxyanthraquinone | 2 | 240.31 | 14864.25 | -103.526 |
| 54 | Alizarin-2-glucoside | 1 | 364.95 | 36746 | -321.115 |
| 55 | Anthraquinone | 0 | 219.3 | 11853.59 | -13.1956 |
| 56 | Juglone | 1 | 185.13 | 8982.661 | -62.3936 |
| 57 | Skikonin | 2 | 274.45 | 22709.22 | -172.017 |
| 58 | Caffeic acid | 2 | 204.55 | 9193.331 | -133.853 |
| 59 | Protocatechuic acid | 2 | 170.13 | 7416.681 | -153.756 |
| 60 | (−)-Epigallocatechin gallate (EGCG) | 8 | 394.2 | 45712.93 | -410.72 |
| 61 | Morin | 5 | 288.48 | 22303.67 | -225.709 |
| 62 | Kaempferol | 4 | 280.24 | 20102.68 | -180.978 |
| 63 | Galangin | 3 | 269.65 | 18478.4 | -135.574 |
| 64 | Phloretin | 4 | 285.23 | 19144.21 | -179.245 |
| 65 | *trans*-chalcone (ck) | 0 | 252.74 | 11463.31 | 28.32257 |
| 66 | Glycitein | 2 | 287.67 | 19644.75 | -132.614 |
| 67 | Genistin | 2 | 388.06 | 41197 | -368.828 |
| 68 | Piceatannol | 4 | 270.78 | 14725.04 | -118.71 |
| 69 | Resveratrol-3- glucoside | 2 | 387.77 | 34687.79 | -294.005 |
| 70 | Scopoletin | 1 | 206.4 | 10777.85 | -118.921 |
| 71 | 5-Methoxyfuranocoumarin | 0 | 220.58 | 13060.76 | -90.7261 |
| 72 | Secoisolariciresinol | 2 | 375.45 | 32205.03 | -230.051 |
| 73 | Pseudopurpurin | 3 | 267.95 | 22203.32 | -242.021 |
| 74 | Chrysazine | 2 | 233.04 | 15390.73 | -115.326 |
| 75 | Ruberythric acid | 1 | 457.65 | 60116.08 | -495.496 |

^a^ n(OH): Number of OH groups

^b^ CA: Cosmo Area

^c^ CCR: Core-Core Repulsion

^d^ FHF: Final Heat of Formation

**Figure** S1. RMSE in tenfold-CV *vs* C and ε with (A) LKF (C=1–500, step=10; ε=0.01–0.1, step=0.01), (B) PKF (C=1–500, step=10; ε=0.01–0.1, step=0.01), and (C) RBF kernel function (C=1–500, step=10, ε=0.01–0.1, step=0.02), (D) g and ε with RBF kernel function (g=0.5–1.5, step=0.1; ε=0.01–0.1, step=0.02), (E) C and g with RBF kernel function (C=1–500, step=10; g=0.5–1.5, step=0.1).
